# Targeting anandamide metabolism rescues core and associated autistic-like symptoms in rats prenatally exposed to valproic acid

**DOI:** 10.1038/tp.2016.182

**Published:** 2016-09-27

**Authors:** M Servadio, F Melancia, A Manduca, A di Masi, S Schiavi, V Cartocci, V Pallottini, P Campolongo, P Ascenzi, V Trezza

**Affiliations:** 1 Department of Science, Section of Biomedical Sciences and Technologies, University “Roma Tre”, Rome, Italy; 2 Department of Physiology and Pharmacology, Sapienza University of Rome, Rome, Italy

## Abstract

Autism spectrum disorders (ASD) are characterized by altered sociability, compromised communication and stereotyped/repetitive behaviors, for which no specific treatments are currently available. Prenatal exposure to valproic acid (VPA) is a known, although still underestimated, environmental risk factor for ASD. Altered endocannabinoid activity has been observed in autistic patients, and endocannabinoids are known to modulate behavioral traits that are typically affected in ASD. On this basis, we tested the hypothesis that changes in the endocannabinoid tone contribute to the altered phenotype induced by prenatal VPA exposure in rats, with focus on behavioral features that resemble the core and associated symptoms of ASD. In the course of development, VPA-exposed rats showed early deficits in social communication and discrimination, compromised sociability and social play behavior, stereotypies and increased anxiety, thus providing preclinical proof of the long-lasting deleterious effects induced by prenatal VPA exposure. At the neurochemical level, VPA-exposed rats displayed altered phosphorylation of CB1 cannabinoid receptors in different brain areas, associated with changes in anandamide metabolism from infancy to adulthood. Interestingly, enhancing anandamide signaling through inhibition of its degradation rescued the behavioral deficits displayed by VPA-exposed rats at infancy, adolescence and adulthood. This study therefore shows that abnormalities in anandamide activity may underlie the deleterious impact of environmental risk factors on ASD-relevant behaviors and that the endocannabinoid system may represent a therapeutic target for the core and associated symptoms displayed by autistic patients.

## Introduction

Autism spectrum disorders (ASD) are a group of severe developmental psychiatric disorders emerging in the early life, for which no specific treatments are currently available. ASD are characterized by altered social interaction, compromised verbal and nonverbal communication, stereotyped and repetitive behaviors,^1^ often associated with comorbid features,^2, 3, 4^ such as social and generalized anxiety.^5, 6, 7^

Both genetic and environmental factors are involved in the etiology of ASD.^8, 9^ Environmental factors include a broad range of influences, such as prenatal exposure to drugs, environmental chemicals, infectious agents, or maternal stress.^10^ One of the best examples of known environmental risk factors for ASD is prenatal exposure to the antiepileptic and mood stabilizer drug valproic acid (VPA). When this drug is taken during pregnancy, it can result in children displaying autistic-like features, such as impaired communication, reduced sociability and stereotyped behaviors.^11, 12^ Results from animal models support the clinical data: prenatal VPA exposure in rodents induces autistic-like signs in the offspring, and therefore it has been proposed as a preclinical model of ASD with face and construct validity.^13, 14, 15, 16^ However, the public awareness of the association between prenatal VPA exposure and adverse outcome in the offspring is still limited. Thus, in March 2016, the French Inspection générale des affaires sociales reported at least 450 cases of malformations in children born between 2006 and 2014 from mothers who had taken VPA during pregnancy,^17^ calling for additional research on both environmental risk factors for ASD that can become targets for effective public health risk reduction efforts, and potential treatments for VPA-induced neurobehavioral dysfunctions.

The endocannabinoid system is a unique biological system that affects a wide range of biological processes, including brain development and functioning. It consists of cannabinoid receptors (CB1 and CB2, mainly expressed in the brain and periphery, respectively), their endogenous ligands (endocannabinoids, mainly anandamide and 2-arachidonoylglycerol (2-AG)), and the enzymes for ligand synthesis and degradation.^18, 19, 20, 21^ In the present study, we used the rat model of ASD based on prenatal VPA exposure to test the hypothesis that an altered endocannabinoid tone contributes to the behavioral alterations observed in ASD, for the following reasons: (1) endocannabinoids are key modulators of socio-emotional responses, cognition, seizure susceptibility, nociception and neuronal plasticity,^22, 23, 24, 25^ all of which are affected in ASD; (2) variations in the CB1 cannabinoid receptor gene modulate social reward responsivity in reward-related forebrain areas,^26, 27^ suggesting that subtle changes in endocannabinoid affinity at CB1 receptors owing to these polymorphisms may underlie the deficits in social reward processing observed in ASD; (3) altered endocannabinoid activity has been observed both in genetic^28, 29, 30, 31^ and environmental^32^ models of ASD and in ASD patients.^33^

We first determined whether the autistic-like behavioral changes exhibited through development by rats prenatally exposed to VPA were associated with changes in the activity of CB1 cannabinoid receptors in brain areas involved in socio-emotional functioning. The endocannabinoid anandamide has a positive modulatory role in key behaviors that are altered in ASD, such as social communication, social play and anxiety-like behaviors,^34, 35, 36, 37, 38, 39^ and it has recently been shown that pharmacological enhancement of anandamide levels corrected the social deficits observed in two genetic models of ASD.^40^ Therefore, we investigated whether prenatal VPA exposure induced changes in brain anandamide synthesis and metabolism, and whether pharmacological manipulation of the endocannabinoid system through modulation of anandamide metabolism was able to restore the altered phenotype displayed by VPA-exposed rats from infancy to adulthood, with focus on behavioral features that resemble the core and associated symptoms of ASD.

## Materials and methods

### Animals

Female Wistar rats (Charles River, Arbresle, France), weighing 250±15 g, were mated overnight. The morning when spermatozoa were found was designated as gestational day 1. Pregnant rats were singly housed in Macrolon cages (40 (length) × 26 (width) × 20 (height) cm), under controlled conditions (temperature 20–21 °C, 55–65% relative humidity and 12/12 h light cycle with lights on at 0700 h). Food and water were available *ad libitum*. On gestational day 12.5, females received a single intraperitoneal injection of either sodium valproate (VPA) or saline (SAL). Newborn litters found up to 1700 h were considered to be born on that day (postnatal day (PND) 0). On PND 1, the litters were culled to eight animals (six males and two females), to reduce the litter size-induced variability in the growth and development of pups during the postnatal period. On PND 21, the pups were weaned and housed in groups of three. The experiments were carried out on the male offspring during infancy (PNDs 9 and 13), adolescence (PND 35) and adulthood (PND 90). One pup per litter from different litters per treatment group was used in each experiment. For every experiment, the exact sample size (*n*) for each experimental group/condition is indicated in the figure legends. The sample size was based on our previous experiments and power analysis.

The experiments were performed in Wistar rats because previous behavioral studies performed with the VPA preclinical model of autism used this rat strain.^15^ Furthermore, we focused on behavioral features, resembling the core and associated symptoms of ASD, that are well documented and can be easily studied in the Wistar rat strain, either from an ethological or pharmacological point of view.

The experiments were approved by the Italian Ministry of Health (Rome, Italy) and performed in agreement with the guidelines released by the Italian Ministry of Health (D.L. 26/14) and the European Community Directive 2010/63/EU.

### Drugs

VPA (Cayman Chemical, Ann Arbor, MI, USA) was dissolved in saline at a concentration of 250 mg ml^−1^ and administered at a dose (500 mg kg^−1^) and time (gestational day 12.5) that have been shown to induce autistic-like behavioral changes in the offspring.^41^

The anandamide hydrolysis inhibitor URB597 (Sigma-Aldrich, Milan, Italy) was dissolved in 5% Tween 80/5% polyethylene glycol/saline and administered intraperitoneally either 2 h (for the behavioral tests performed at adolescence and adulthood) or 30 min (for the behavioral tests performed at infancy) before testing. Drug dose and pre-treatment intervals were based on literature,^29, 35, 37, 38, 42^ and on pilot experiments. The solutions were administered in a volume of 2.5 ml kg^−1^ at infancy, 2 ml kg^−1^ at adolescence and 1 ml kg^−1^ at adulthood.

### Western blot analysis of phosphorylated and total CB1 cannabinoid receptor

The rats were rapidly decapitated and their brains removed and cut into coronal slices on a cold plate. The prefrontal cortex, dorsal striatum, nucleus accumbens, hippocampus, amygdala and cerebellum were dissected by hand under microscopic control within 2 min. The tissues were stored at −80 °C until use.

Lysates from the tissues were homogenized in a homogenization buffer (0.01 m Tris-HCl, 0.001 m CaCl_2_, 0.15 m NaCl, 0.001 m PMSF (phenylmethylsulfonyl fluoride), pH 7.5) w/v 1:10 (ref. 43) by sonication. An aliquot of homogenate was resuspended in sample buffer (0.250 m Tris-HCl—pH 6.8—containing 20% SDS, 0.001 m PMSF). Lysate samples were boiled for 5 min before loading to the SDS-PAGE (polyacrylamide gel electrophoresis).

Proteins (20 μg) from lysate samples were resolved by 7% SDS-PAGE at 30 mA (constant current) for 120 min and blotted to nitrocellulose membranes (Bio-Rad, Hercules, CA, USA). Immunoblots were incubated with primary antibodies anti P-CB1 and CB1 (1:500; Santa Cruz Biotechnology, Santa Cruz, CA, USA, Catalog No. sc-17555 and sc-10068), followed by secondary peroxidase-conjugated antibodies (1:3000; Santa Cruz Biotechnology, Catalog No. sc-2020). Immunoreactivity was detected by enhanced chemiluminescence (GE Healthcare, Little Chalfont, UK). The nitrocellulose membrane was stripped with Restore western blot stripping buffer (Pierce Chemical, Rockford, IL, USA) for 15 min at room temperature and re-probed with anti-tubulin (Sigma, Catalog No. T9026) antibody. Bound antibodies to proteins onto nitrocellulose were visualized by using enhanced chemoluminescence detection (GE Healthcare) and exposure to Amersham Hyperfilm ECL (GE Healthcare). The images were analyzed with ImageJ (National Institutes of Health).

### Quantitative PCR analysis of fatty acid amide hydrolase and *N*-acylphosphatidylethanolamines-phospholipid D expression

Two micrograms of the total RNA extracted from the whole rat brain were reverse-transcribed using an oligo-dT primer to prime the reverse transcription and the SuperScript II Reverse Transcriptase system (Life Technologies). The quantitative PCR (qPCR) of fatty acid amide hydrolase (FAAH) and *N*-acylphosphatidylethanolamines-phospholipid D (NAPE-PLD) was performed using a Rotor-Gene 6000 thermal cycling system (Corbett Research, Mortlake, NSW, Australia) and the Hydra SYBR qPCR Master Mix (BioLab, Rome, Italy) as detection system. The thermal cycling conditions for all the transcripts analyzed were: 95 °C for 2 min and 40 cycles at 95 °C for 10 s and 60 °C for 30 s. PCR primers were the following: FAAH_FW: 5'-TGCTGAAGCCTCTGTTTCCT-3' FAAH_RV: 5'-TCTCATGCTGCAGTTTCCAC; NAPE_FW: 5'-TCGCTGGGGATACTGGTTAC-3' NAPE_RV: 5'-AAGCTCCAATGGGAATAGCC; β-ACTIN_FW: 5'-AGGCCATGTACGTAGCCATCCA-3' β-ACTIN_RV: 5'-TCTCCGGAGTCCATCACAATG-3'. The fold changes of FAAH and NAPE transcripts expression in VPA- vs SAL-treated samples were calculated using the 2^–ΔΔCT^ method.

### Reproduction data

Body weights of the dams were taken daily throughout pregnancy and the length of pregnancy was determined. Litter size, male/female ratio, weight gain of pups and postnatal vitality were also measured.

### Isolation-induced ultrasonic vocalizations

On PND 9, the pups were individually placed into a Plexiglas arena (30 (length) × 30 (width) × 30 (height) cm), located inside a sound-attenuating and temperature-controlled chamber, with a camera positioned above the arena. The ultrasonic vocalizations (USVs) emitted by the pup were detected for 3 min by an ultrasound microphone (Avisoft Bioacoustics, Glienicke, Germany) sensitive to frequencies between 10 and 200 kHz. Pup axillary temperature was measured before and after the test by a digital thermometer. The USVs were analyzed, both quantitatively and qualitatively, using Avisoft Recorder software (Version 5.1), and classified in six different groups according to number of syllables, frequency modulation and duration:^44, 45, 46^ flat calls (calls with constant frequency with a maximum variation of ±5 kHz); up/downward (single syllable calls emitted at ±5 kHz with a single frequency modulation); flat high frequency calls (calls with similar constant frequency, but emitted at >75 kHz); syllable calls (calls composed by two or more syllables); complex calls (calls displaying complex frequency modulation); short calls (calls with durations shorter than 5 ms).

### Homing behavior

On PND 13, the litter was separated from the dam and kept for 30 min in a temperature-controlled holding cage. Then, each pup was placed into a Plexiglas box whose floor was one-third covered with bedding from the pup's home cage and two-third with clean bedding. The pup was located at the side of the box covered by clean bedding, and its behavior was videorecorded for 4 min for subsequent analysis. The following parameters were scored using the Observer 3.0 software (Noldus Information Technology, Wageningen, The Netherlands): latency (seconds) to reach the home-cage bedding area; total time (seconds) spent by the pup in the nest bedding area; total number of entries into the nest bedding area.^45^

### Three-chamber test

The apparatus was a rectangular three-chamber box, with two lateral chambers (30 (length) × 35 (width) × 35 (height) cm) connected to a central chamber (15 (length) × 35 (width) × 35 (height) cm). Each lateral chamber contained a small Plexiglas cylindrical cage. The test was performed as previously described.^47^ Each experimental rat was individually allowed to explore the apparatus for 10 min, and then confined in the central compartment. An unfamiliar stimulus animal was placed into the Plexiglas cage in one chamber of the apparatus, whereas the cage in the other chamber was left empty. Both doors to the side chambers were then opened, allowing the experimental animal to freely explore the apparatus for 10 min. The percent of time spent in social approach (sniffing the stimulus and the cage confining it) was scored using the Observer 3.0 software (Noldus Information Technology).

### Social play behavior

Rats were individually habituated to the test cage for 10 min on each of the 2 days before testing, as previously described.^37, 42, 48^ On the test day, the animals were isolated for 3 h before testing.^49, 50, 51^ The test consisted of placing VPA- or SAL-exposed rats together with an untreated animal for 15 min. The behavior was assessed for each individual animal of a pair separately using the Observer 3.0 software (Noldus Information Technology). The behaviors of the animals were recorded using a camera with zoom lens, video tape recorder and television monitor.

In rats, a bout of social play behavior starts with one rat soliciting (‘pouncing') another animal, by attempting to nose or rub the nape of its neck. The animal that is pounced upon can respond in different ways: if the animal fully rotates to its dorsal surface, ‘pinning' is the result (one animal lying with its dorsal surface on the floor with the other animal standing over it), which is considered the most characteristic posture on social play behavior in rats.^52^

We determined ‘play responsiveness' (that is, the percentage of response to play solicitation) as the probability of an animal of being pinned in response to play solicitation (pouncing) by the stimulus partner.^42^

### Elevated plus maze

The apparatus comprised two open and two closed arms (50 (length) × 10 (width) × 40 (height) cm) that extended from a common central platform (10 (length) × 10 (width) cm). The rats were individually placed on the central platform of the maze for 5 min. Each session was recorded with a camera positioned above the apparatus for subsequent behavioral analysis performed using the Observer 3.0 software (Noldus Information Technology). The following parameters were analyzed:^53^

1. % time spent in the open arms (% TO): (seconds spent on the open arms of the maze/seconds spent on the open+closed arms) × 100;
2. % open-arm entries (% OE): (the number of entries into the open arms of the maze/number of entries into open+closed arms) × 100;
3. Number of closed-arm entries (number of CE).

### Hole-board test

The apparatus was a gray square metal table (40 (length) × 40 (width) × 10 (height) cm) with 16 evenly spaced holes (4 cm in diameter), inserted in a Plexiglas arena (40 (length) × 40 (width) × 60 (height) cm). The rats were individually placed in the apparatus and their behavior was observed for 5 min. Dipping behavior was scored by the number of times an animal inserted its head into a hole at least up to the eye level. Each session was recorded with a camera positioned above the apparatus for subsequent behavioral analysis performed using the Observer 3.0 software (Noldus Information Technology).

### Statistical analysis

The data are expressed as mean±s.e.m. To assess the effects of the prenatal treatment (VPA or SAL) on the offspring, data were analyzed with Student's *t*-tests. Two-way analysis of variance (ANOVA) was used to assess the effects of prenatal and postnatal treatments, using prenatal (VPA or SAL) and postnatal (URB597 or vehicle) treatments as between-subjects factor. Two-way ANOVA was followed by Student's–Newman–Keuls *post hoc* test where appropriate.

The western blot and qPCR experiments were performed in duplicate and the results were comparable.

Behavioral experiments were scored and analyzed by a trained observer who was unaware of the treatment conditions (Noldus Information Technology). Similarly, biochemical experiments were performed and analyzed in blinded conditions.

Supplementary Figure 1 shows three different timeline diagrams for: (1) the behavioral characterization of the VPA- and SAL-exposed offspring; (2) the behavioral effects of URB597 in the VPA- and SAL-exposed offspring; (3) the western blot and quantitative PCR analyses of the brain of VPA- and SAL-exposed animals.

## Results

### Reproduction data

During gestation, no differences in body weight gains were observed between VPA- and SAL-treated dams. Prenatal VPA exposure did not affect pregnancy length, litter size at birth, male/female ratio, pup weight gain and postnatal vitality (Supplementary Table 1).

### CB1 cannabinoid receptor phosphorylation and FAAH and NAPE-PLD expression

**Figure 1:**
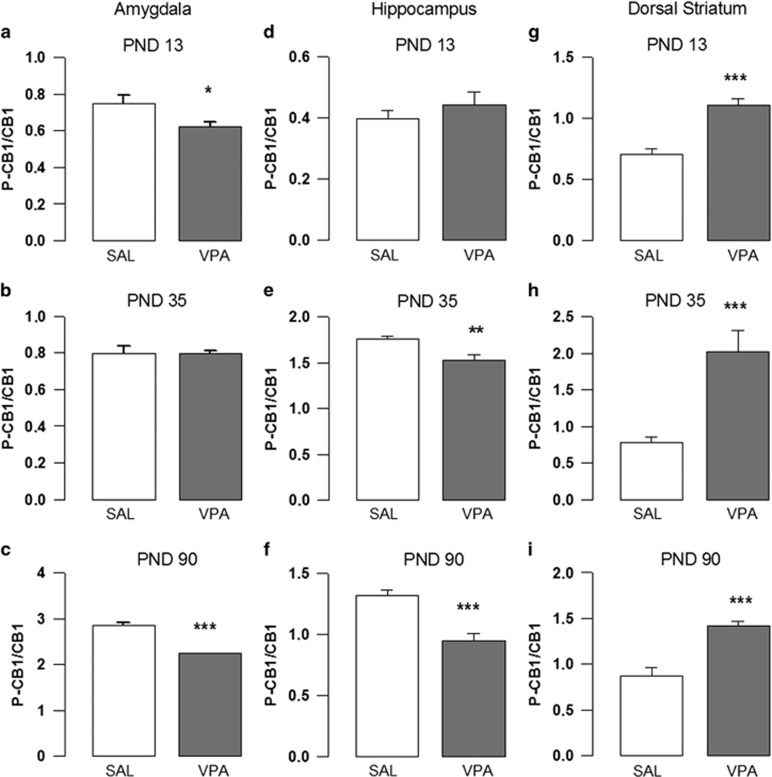
Activation of CB1 receptors. Ratio between phosphorylated and total CB1 receptor protein in the amygdala (**a**–**c**), hippocampus (**d**–**f**) and dorsal striatum (**g**–**i**) of VPA- and SAL-exposed offspring, evaluated at PNDs 13 (*n* (SAL)=6, *n* (VPA)=6), 35 (*n* (SAL)=6, *n* (VPA)=6) and 90 (*n* (SAL)=6, *n* (VPA)=6). Data represent mean±s.e.m.; *P<0.05; **P<0.01; ***P<0.001 (Student's *t*-test). PND, postnatal day; SAL, saline; VPA, valproic acid.

Phosphorylation may reflect the activation of CB1 cannabinoid receptors.^54^ To investigate whether VPA prenatal exposure induced changes in the activity of brain cannabinoid receptors, we measured both phosphorylated and total CB1 receptor protein expression in the prefrontal cortex, dorsal striatum, nucleus accumbens, hippocampus, amygdala and cerebellum of the offspring at PNDs 13, 35 and 90. In VPA-exposed rats, the ratio between phosphorylated and total CB1 receptor protein decreased in the amygdala at PND 13 (*t*=2.103, *P*<0.05, df=10; Figure 1a) and 90 (*t*=11.41, *P*<0.001, df=10; Figure 1c) but not at PND 35 (*t*=0.06718, *P*=NS (not significant), df=10) and in the hippocampus at PND 35 (*t*=3.439, *P*<0.01, df=10; Figure 1e) and PND 90 (*t*=4.802, *P*<0.001, df=10; Figure 1f), but not at PND 13 (*t*=1.369, *P*=NS, df=10). The phosphorylation state of CB1 receptors increased in the dorsal striatum of VPA-exposed rats at all ages (PND 13: *t*=6.056, *P*<0.001, df=10; Figure 1g; PND 35: *t*=4.082, *P*<0.001, df=10; Figure 1h; PND 90: *t*=5.601, *P*<0.001, df=10; Figure 1i). No differences between VPA- and SAL-exposed rats were observed in the cerebellum (PND 13: *t*=0.8856, *P*=NS, df=10; PND 35: *t*=0.05827, *P*=NS, df=10; PND 90: *t*=0.8579, *P*=NS, df=10), prefrontal cortex (PND 13: *t*=1.897, *P*=NS, df=10; PND 35: *t*=0.1135, *P*=NS, df=10; PND 90: *t*=0.8790, *P*=NS, df=10) and nucleus accumbens (PND 13: *t*=0.3687, *P*=NS, df=10; PND 35: *t*=0.1578, *P*=NS, df=10; PND 90: *t*=1.365, *P*=NS, df=10). Significant changes in CB1 total receptor levels were only observed in the striatum of adolescent (*t*=5.88, *P*=NS, df=10) and adult (*t*=2.47, *P*=NS, df=10) rats and in the hippocampus of adult animals (*t*=4.15, *P*=NS, df=10).

**Figure 2:**
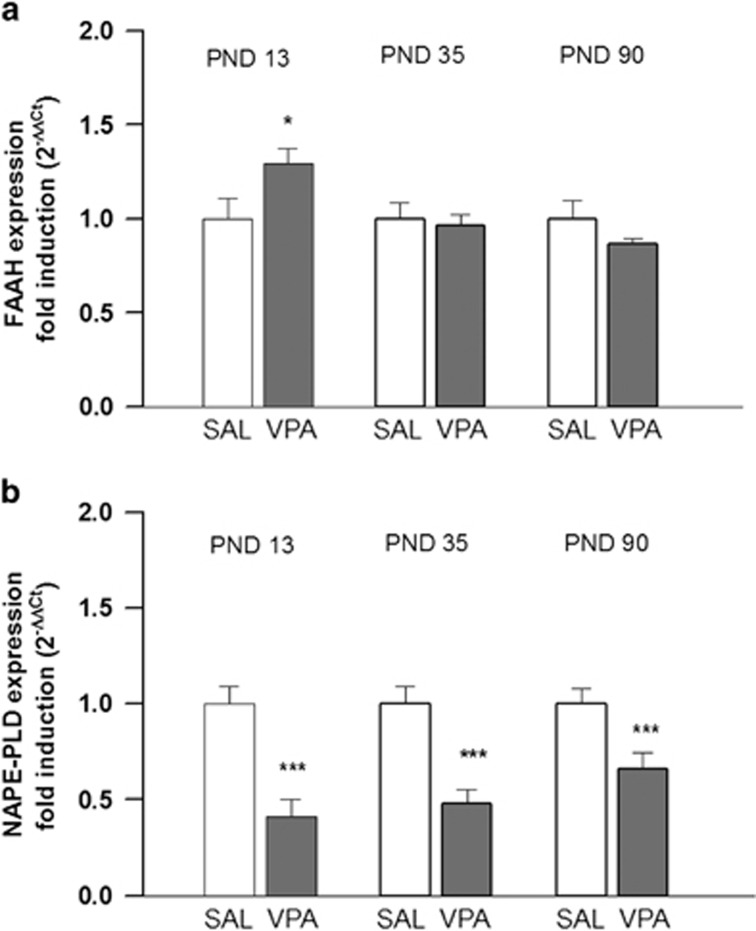
FAAH and NAPE-PLD expression. Fold induction of FAAH (**a**) and NAPE-PLD (**b**) expression in the brain of VPA- and SAL-exposed offspring, evaluated at PNDs 13 (*n* (SAL)=3, *n* (VPA)=3), 35 (*n* (SAL)=3, *n* (VPA)=3) and 90 (*n* (SAL)=3, *n* (VPA)=3). Data represent mean±s.d.; *P<0.05; ***P<0.001 (Student's *t*-test). FAAH, fatty acid amide hydrolase; NAPE-PLD, *N*-acylphosphatidylethanolamines-phospholipid D; PND, postnatal day; SAL, saline; VPA, valproic acid.

We next evaluated the expression of FAAH, which catalyzes anandamide hydrolysis, and NAPE-PLD, which catalyzes the one-step conversion of NAPEs to anandamide, in the brain of the offspring at PNDs 13, 35 and 90. In VPA-exposed animals, FAAH expression was increased at PND 13 (*t*=3.8832, *P*<0.05, df=4) and unchanged at PNDs 35 (*t*=0.6928, *P*=NS, df=4) and 90 (*t*=2.6943, *P*=NS, df=4; Figure 2a). NAPE-PLD expression was decreased in VPA-exposed animals at all ages (PND 13: *t*=11.2583, *P*<0.001, df=4; PND 35: *t*=18.0133, *P*<0.001, df=4; PND 90: *t*=11.1207, *P*<0.001, df=4; Figure 2b).

### Isolation-induced USVs

**Figure 3:**
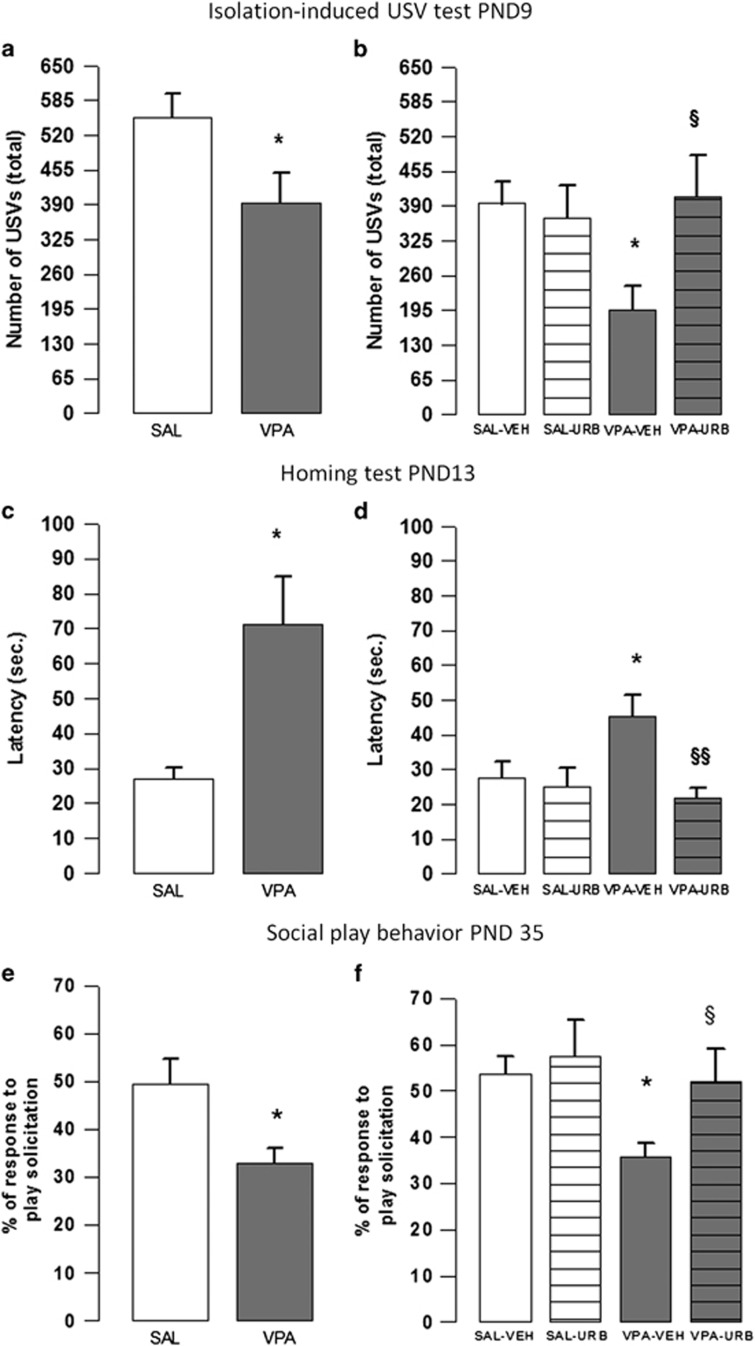
Isolation-induced USV, homing and social play behavior tests. Prenatal VPA exposure reduced isolation-induced USV emission at PND 9 (**a**; *n* (SAL)=13, *n* (VPA)=17), altered homing behavior at PND 13 (**c**; *n* (SAL)=10, *n* (VPA)=15) and reduced social play behavior at PND 35 (**e**; *n* (SAL)=14, *n* (VPA)=12). URB597 reversed the altered behavioral phenotype displayed by VPA-exposed rats in the isolation-induced USV (**b**; *n* (SAL-VEH)=12, *n* (VPA-VEH)=11, *n* (SAL-URB)=10, *n* (VPA-URB)=9), homing (**d**; *n* (SAL-VEH)=11, *n* (VPA-VEH)=11, *n* (SAL-URB)=14, *n* (VPA-URB)=8) and social play behavior (**f**; *n* (SAL-VEH)=9, *n* (VPA-VEH)=11, *n* (SAL-URB)=8, *n* (VPA-URB)=8) tests. Data represent mean±s.e.m. *P<0.05 vs SAL-VEH group; ^§^*P*<0.05, ^§§^*P*<0.01 vs VPA-VEH group (Student's *t*-test (**a**, **c**, **e**); Student's–Newman–Keuls *post hoc* test (**b**, **d**, **f**)). PND, postnatal day; SAL, saline; USV, ultrasonic visualization; VEH, vehicle; VPA, valproic acid.

When separated from the dam and siblings on PND 9, VPA-exposed pups vocalized significantly less compared with SAL-exposed pups (*t*=2.15, *P*<0.05, df=28; Figure 3a). No differences between the two groups were found in the percentage of the different call categories emitted: flat (*t*=0.82, *P*=NS, df=14); flat high frequency (*t*=1.54, *P*=NS, df=14); syllable (*t*=0.69, P=NS, df=14); short (*t*=1.08, *P*=NS, df=14); up/downward (*t*=0.67, *P*=NS, df=14); complex (*t*=0.04, *P*=NS, df=14). URB597 reversed the communicative deficits displayed by VPA-exposed animals, without altering the performance of SAL-exposed pups (Figure 3b). A two-way ANOVA analysis performed on the number of total USVs emitted after treatment with URB597 or vehicle gave the following results: total USVs (F_(prenatal treat.)1,38_=2.021, *P*=NS; F_(treat.)1,38_=2.741, *P*=NS; F_(prenatal treat. × treat.)1,38_=4.586; *P<*0.05). *Post hoc* analysis revealed that VPA-exposed pups emitted less USVs compared with SAL-exposed pups (*P*<0.05). However, VPA-exposed pups treated with URB597 did not differ from SAL-exposed pups treated with either VEH (*P*=NS) or URB (*P*=NS; Figure 3b).

### Homing behavior

The VPA-exposed rats showed deficits in the homing test on PND 13: they showed longer latency to reach the home-cage bedding (*t*=2.55, *P*<0.05, df=23; Figure 3c) and spent less time in the nest area (*t*=2.55, *P*<0.05, df=23; not shown) compared with SAL-exposed offspring. The two groups did not differ in locomotor activity (number of crossings: *t*=0.90, *P*=NS, df=23). URB597 reversed the deficit displayed by VPA-exposed pups in this test, without altering the performance of SAL-exposed pups. A two-way ANOVA analysis performed on the parameters measured in the test gave the following results: latency (F_(prenatal treat.)1,40_=1.77, *P*=NS; F_(treat.)1,40_=5.45, *P*<0.05; F_(prenatal treat. × treat.)1,40_=3.53; *P*=NS); nesting time (F_(prenatal treat.)1,40_=1.34, *P*=NS; F_(treat.)1,40_=6.72, *P*<0.05; F_(prenatal treat. × treat.)1,40_=7.11; *P*<0.05). *Post hoc* analysis revealed that VPA-exposed pups showed a longer latency to reach the familiar bedding (*P<*0.05; Figure 3d) and spent less time in the nest area (*P*<0.01, not shown) compared with SAL-exposed pups. Conversely, no differences were found between VPA-exposed pups treated with URB597 and SAL-exposed pups treated with vehicle.

### Social play behavior

The VPA-exposed rats showed reduced play responsiveness compared with SAL-exposed animals (*t*=2.56, *P*<0.05, df=24; Figure 3e). When VPA- and SAL-exposed animals were treated with URB597 or its vehicle, a two-way ANOVA analysis performed on the percentage of response to play solicitation gave the following results: (F_(prenatal treat.)1,32_=4.42, *P*<0.05; F_(treat.)1,32_=4.52, *P*<0.05; F_(prenatal treat. × treat.)1,32_=1.2; *P*=NS). *Post hoc* analysis revealed that VPA-exposed rats responded less to social play solicitation (*P*<0.05; Figure 3f) compared with SAL-exposed rats. Conversely, no differences were found between VPA-exposed rats treated with URB597 and SAL-exposed rats treated with vehicle.

### Three-chamber test

**Figure 4:**
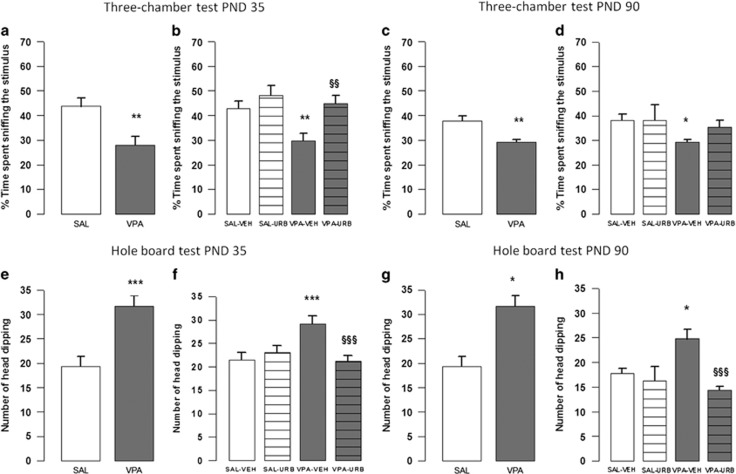
Three-chamber and hole-board tests. Prenatal VPA exposure reduced sociability in the three-chamber test (**a** and **c**) at PND 35 (*n* (SAL)=8, *n* (VPA)=9) and 90 (*n* (SAL)=15, *n* (VPA)=13) and induced stereotypic behavior in the hole-board test (**e** and **g**) at PND 35 (*n* (SAL)=11, *n* (VPA)=12) and 90 (*n* (SAL)=12, *n* (VPA)=12). URB597 mitigated the altered sociability (**b** and **d**) displayed by VPA-exposed offspring at PND 35 (*n* (SAL-VEH)=13, *n* (VPA-VEH)=13, *n* (SAL-URB)=8, *n* (VPA-URB)=10) and 90 (*n* (SAL-VEH)=11, *n* (VPA-VEH)=12, *n* (SAL-URB)=8, *n* (VPA-URB)=8). Furthermore, URB597 mitigated the stereotypies (**f** and **h**) displayed by VPA-exposed offspring at PND 35 (*n* (SAL-VEH)=20, *n* (VPA-VEH)=19, *n* (SAL-URB)=20, *n* (VPA-URB)=19) and 90 (*n* (SAL-VEH)=8, *n* (VPA-VEH)=10, *n* (SAL-URB)=8, *n* (VPA-URB)=8). Data represent mean±s.e.m. *P<0.05, **P<0.01, ***P<0.001 vs SAL-VEH group; ^§§^*P*<0.01, ^§§§^*P*<0.01 vs VPA-VEH group (Student's *t*-test (**a**, **c**, **e** and **g**); Student's–Newman–Keuls *post hoc* test (**b**, **d**, **f** and **h**)). PND, postnatal day; SAL, saline; VEH, vehicle; VPA, valproic acid.

Compared with the SAL-exposed animals, the VPA-exposed rats showed decreased sociability in the three-chamber test, as they spent less time sniffing the stimulus animal both at PNDs 35 (*t*=3.14, *P*<0.01, df=15; Figure 4a) and 90 (*t*=3.37, *P*<0.01, df=26; Figure 4c). No differences between the two groups were found in the total number of entries (PND 35: *t*=0.13, *P*=NS, df=15; PND 90: *t*=1.44, *P*=NS, df=26, not shown). When VPA- and SAL-exposed animals were treated with URB597 or its vehicle, a two-way ANOVA analysis performed on the percentage of time spent sniffing the stimulus gave the following results: PND 35 (F_(prenatal treat.)1,40_=6.05, *P*<0.05; F_(treat.)1,40_=9.31, *P*<0.01; F_(prenatal treat. × treat.)1,40_=2.1; *P*=NS); PND 90 (F_(prenatal treat.)1,35_=4.13, *P*<0.05; F_(treat.)1,35_=1.07, *P*=NS; F_(prenatal treat. × treat.)1,35_=1.1; *P*=NS). *Post hoc* analysis revealed that VPA-exposed rats spent less time sniffing the stimulus than SAL-exposed rats both at PND 35 (*P*<0.01; Figure 4b) and PND 90 (*P*<0.05; Figure 4d). Conversely, no differences were found between VPA-exposed rats treated with URB597 and animals SAL-exposed rats treated with vehicle.

### Hole-board test

The VPA-exposed rats showed stereotypic behaviors in the hole-board test, as they made more head dippings at PND 35 (*t*= 4.14, *P*<0.001, d.f.=21; Figure 4e) and PND 90 (*t*=2.27, *P*<0.05, df=22; Figure 4g). URB597 reduced the stereotypic behavior found in VPA-exposed rats. A two-way ANOVA analysis performed on the number of head dippings gave the following results: PND 35 (F_(prenatal treat.)1,74_=3.93, *P*=NS; F_(treat.)1,74_=4.6, *P*<0.05; F_(prenatal treat. × treat.)1,74_=10.49; *P*<0.01); PND 90 (F_(prenatal treat.)1,30_=1.32, *P*=NS; F_(treat.)1,30_=10.67, *P*<0.01; F_(prenatal treat. × treat.)1,30_=4.82; *P*<0.05). *Post hoc* analysis revealed that VPA-exposed adolescent (*P*<0.001; Figure 4f) and adult (*P*<0.05; Figure 4h) rats made more head dippings than SAL-exposed rats. Conversely, no differences were found between VPA-exposed rats treated with URB597 and SAL-exposed rats treated with vehicle.

### Elevated plus maze test

**Figure 5:**
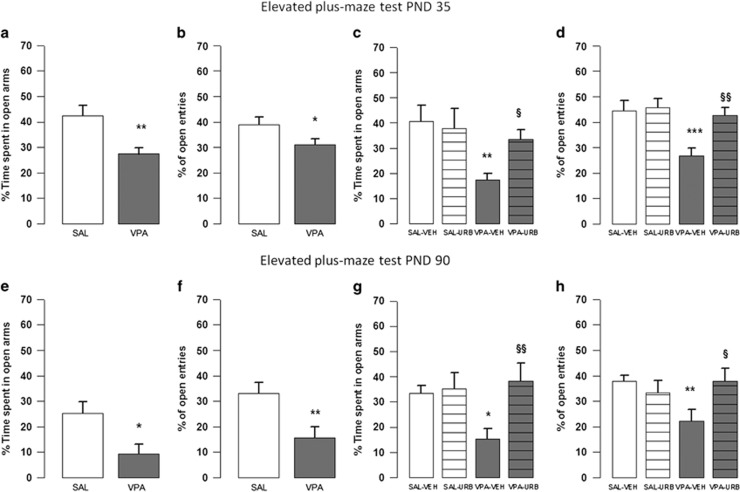
Elevated plus maze test. Prenatal VPA exposure decreased the percentage of time spent by the offspring in the open arms (**a** and **e**) and the percentage of open-arm entries (**b** and **f**) in the elevated plus maze test at PND 35 (*n* (SAL)=23, *n* (VPA)=21) and 90 (*n* (SAL)=13, *n* (VPA)=10). URB597 reversed the anxiety-like behavior displayed by VPA-exposed rats (**c**, **d**, **g** and **h**) at PND 35 (*n* (SAL-VEH)=11, *n* (VPA-VEH)=11, *n* (SAL-URB)=8, *n* (VPA-URB)=8) and 90 (*n* (SAL-VEH)=17, *n* (VPA-VEH)=15, *n* (SAL-URB)=13, *n* (VPA-URB)=13). Data represent mean±s.e.m. *P<0.05, **P<0.01, ***P<0.001 vs SAL-VEH group; ^§^*P*<0.05, ^§§^*P*<0.01 vs VPA-VEH group (Student's *t*-test (**a-f**); Student's–Newman–Keuls *post hoc* test (**c**–**h**)). PND, postnatal day; SAL, saline; VEH, vehicle; VPA, valproic acid.

Both at PNDs 35 and 90, the VPA-exposed rats showed an anxious-like phenotype compared with SAL-exposed animals, as they spent less time in the open arms (PND 35: *t*=3.18, *P*<0.01, df=42; Figure 5a; PND 90: *t*=2.55, *P*<0.05, df=21; Figure 5e) and made less open-arm entries (PND 35: *t*=2.08, *P*<0.05, df=42; Figure 5b; PND 90: *t*=2.86, *P*<0.01, df=21; Figure 5f) compared to SAL-exposed rats. No significant differences have been found in the total number of closed-arm entries either at PND 35 (*t*=1.64, *P*=NS, df=42, data not shown) or 90 (*t*=0.65, *P*=NS, df=21, data not shown). URB597 had anxiolytic-like effects in VPA-exposed rats, without altering the behavior of SAL-exposed animals. A two-way ANOVA analysis performed on the parameters measured in this test gave the following results: PND 35, percentage of time spent in the open arms (F_(prenatal treat.)1,34_=6.09, *P*<0.05; F_(treat.)1,34_=1.41, *P*=NS; F_(prenatal treat. × treat.)1,34_=2.87, *P*=NS); percentage of open-arm entries (F_(prenatal treat.)1,34_=8.60, *P*<0.01; F _(treat.)1,34_=5.93, *P*<0.05; F_(prenatal treat. × treat.)1,34_=4.51, *P*<0.05); PND 90, percentage of time spent in the open arms (F_(prenatal treat.)1,54_=2.12, *P*=NS; F_(treat.)1,54_=5.79, *P*<0.05; F_(prenatal treat. × treat.)1,54_=8.08, *P*<0.05; Figure 5g); percentage of open-arm entries (F_(prenatal treat.)1,54_=1.76, *P*=NS; F_(treat.)1,54_=1.85, *P*=NS; F_(prenatal treat. × treat.)1,54_=5.84, *P*<0.05; Figure 5h). *Post hoc* analysis showed that VPA-exposed animals spent less time in the open arms and made less open-arm entries compared with SAL-exposed rats (Figures 5c and d, g and h). Conversely, no differences were found between VPA-exposed animals treated with URB597 and SAL-exposed animals treated either with vehicle or with URB. Last, a two-way ANOVA analysis performed on the total number of closed-arm entries gave the following results: adolescence (F_(prenatal treat.)1,34_=1.2, *P*=NS; F_(treat.)1,34_=0.45, *P*=NS; F_(prenatal treat. × treat.)1,34_=0.06, *P*=NS); adulthood (F_(prenatal treat.)1,54_=1.1, *P*=NS; F_(treat.)1,54_=0.36, *P*=NS; F_(prenatal treat. × treat.)1,54_=0.17, *P*=NS).

## Discussion

Endocannabinoids are known to regulate key brain functions that are altered in ASD. By using a well-validated animal model of ASD based on prenatal VPA exposure in rats,^15, 16^ we provide new evidence that an altered anandamide tone that manifests already at infancy and persists into adolescence and adulthood may underlie core and associated autistic-like symptoms, thus providing preclinical rationale to a potential role of anandamide signaling as a new therapeutic target for ASD.

At the neurochemical level, we found that rats prenatally exposed to VPA display altered expression of phosphorylated CB1 cannabinoid receptors in the amygdala, hippocampus and dorsal striatum, with no changes in the prefrontal cortex, cerebellum and nucleus accumbens. As phosphorylation may reflect the activation of CB1 cannabinoid receptors,^54^ the observed changes may be a compensatory response aimed at normalizing CB1-mediated signaling in VPA-exposed rats as a consequence of a relative imbalance of the system.^55^ To support this possibility, we found changes in the expression of the enzymes that catalyze the synthesis and degradation of anandamide in the brain of infant, adolescent and adult rats prenatally exposed to VPA, indicating that prenatal VPA exposure induces long-lasting changes in brain anandamide metabolism. These results extend previous findings showing changes in the expression of mRNA for the enzymes primarily responsible for the synthesis and degradation of 2-AG in the cerebellum and hippocampus of VPA-exposed rats, with no changes in both 2-AG and anandamide content.^32^

At the behavioral level, our results confirm the validity of prenatal exposure to VPA as an animal model of ASD with strong face validity. In particular, our longitudinal study shows that prenatal VPA exposure induces early communicative deficits, altered sociability and social play behavior, enduring stereotypies and increased anxiety in the rat offspring. At infancy, in line with previous studies,^56, 57, 58, 59^ we found quantitative changes in the USVs emitted by VPA-exposed rats when separated from their mother and siblings compared with control animals. USVs have an essential communicative role in mother–offspring interaction,^60^ and therefore the altered USV profile displayed by VPA-exposed pups when separated from the nest may indicate a reduced ability to communicate with their mother. Furthermore, as previously reported,^15, 58, 61, 62, 63^ we found that VPA-exposed pups were unable to use olfactory cues to discriminate between a neutral odor and their own home cage odor in the homing behavior test. Olfaction, and in particular the learned association between maternal odors and maternal stimulation, is crucial for the development of social behavior and social recognition.^64, 65^ Altogether, the altered USV profile and homing behavior displayed by VPA-exposed pups indicate their profound deficits in social communication and social discrimination since the first days of life.

At adolescence and adulthood, the consequences of prenatal VPA exposure on the social behavior of the offspring were analyzed at two different levels: (1) during a free dyadic social encounter with a same-age stimulus animal, to analyze the reciprocal nature of the social interaction and its ethologic characteristics; (2) in the three-chamber test that focuses on the social approach of the experimental animal toward a confined stimulus animal, without direct social contact.

In accordance with previous studies,^15, 32, 41, 57, 58, 61, 66, 67, 68, 69^ the VPA-exposed rats showed deficits in social behavior both at adolescence and adulthood, as they showed decreased responsiveness to play solicitation and reduced sociability in the three-chamber test. The reduced willingness to engage in social interaction or to prolong a playful encounter displayed by VPA-exposed rats are reminiscent of the altered social behavior and deficient social play observed in autistic patients.^1^

The endocannabinoid system modulates different aspects of social behavior. In humans, variations in the CB1 cannabinoid receptor gene predict differences in the striatal response to happy faces, indicating a role of the endocannabinoid system in social reward responsivity.^26, 27^ In addition, the endocannabinoid system modulates amygdala reactivity to social threat signals.^70^ Altered striatal and amygdala activity has been related to the diminished response to social rewards displayed by autistic patients.^71, 72^ In line with this scenario, we found that VPA-exposed rats show altered phosphorylation of CB1 cannabinoid receptors in the dorsal striatum during all the lifespan, and in the amygdala both at infancy and adulthood.

In rodents, anandamide has a positive modulatory role in several social behavior: it is involved in pup USV production and emotional reactivity,^35, 73^ it is released during social play in the amygdala, nucleus accumbens^36^ and dorsal striatum,^74^ and it is involved in adult forms of social interactions.^34, 75^ Interestingly, we found that the expression of NAPE-PLD, the main anandamide biosynthetic enzyme, was reduced in VPA-exposed rats from infancy to adulthood; furthermore, VPA-exposed infant rats showed higher expression of FAAH, the main enzyme involved in anandamide catabolism. Thus, a reduced anandamide-mediated signaling may underlie the deficits in the communicative and social domain displayed by VPA-exposed rats through development. In line with this possibility, enhancing anandamide activity through administration of the anandamide hydrolysis inhibitor URB597 normalized the USV profile and the performance of VPA-exposed pups in the homing test, and reversed their social deficits in the three-chamber and social play behavior tests.

Stereotyped behaviors are the second core symptom displayed by ASD patients.^1^ In accordance with previous studies,^15, 58, 66, 67, 76^ we found that both adolescent and adult rats prenatally exposed to VPA showed stereotyped behavior in the hole-board test. It is known that the endocannabinoid system is involved in motor control by modulating dopaminergic neurotransmission in the basal ganglia.^77^ In particular, increasing endocannabinoid signaling by inhibiting endocannabinoid uptake or metabolism counteracts the stereotypies induced by systemic or intrastriatal dopaminergic stimulation in rodents,^78, 79, 80^ whereas antagonism at CB1 cannabinoid receptors increases dopamine receptor-mediated stereotypies.^81^ In light of these findings, it is therefore not surprising that the anandamide hydrolysis inhibitor URB597 counteracted the stereotyped behavior displayed by both adolescent and adult VPA-exposed rats.

Anxiety is a frequent symptom displayed by autistic patients.^2^ Accordingly, as previously reported,^41, 66, 82, 83^ the VPA-exposed rats showed an anxious phenotype in the elevated plus maze test, both during adolescence and adulthood.

Animal and clinical studies have repeatedly shown that the endocannabinoid system is critically involved in the control of emotionality.^21^ In particular, URB597 induces anxiolytic-like effects in rodents,^35, 38, 39^ that become evident particularly when the animals are tested under stressful conditions,^84, 85^ supporting the idea that endocannabinoids are released ‘on demand', in response to challenging situations. Interestingly, we found that URB597, administered at a dose that had no effect in control animals, reduced the anxious phenotype displayed by both adolescent and adult VPA-exposed rats.

Collectively, three main conclusions can be drawn from the present study. First, the altered behavioral profile displayed by VPA-exposed rats from infancy till adolescence and adulthood is a further preclinical proof of the long-lasting deleterious effects induced by prenatal VPA exposure. To date, the potential for some environmental factors to promote ASD is still relatively undetermined, and the recent epidemiological data clearly show that the public health protection strategies adopted so far have been inadequate at best.^17^ Thus, targeted research on causative environmental factors for ASD is warranted. Second, we report abnormalities in brain anandamide activity in an environmental animal model of ASD based on prenatal VPA exposure in rats. These findings, that contribute to increase our understanding of the neural underpinnings of ASD, extend previous studies reporting altered endocannabinoid activity in animal models of ASD^28, 29, 30, 31, 32^ and in ASD patients,^33^ and genetic studies in humans showing an important role for cannabinoid receptors in processing appetitive socially relevant stimuli.^26, 27^ Last, our results prove that pharmacological interference with anandamide metabolism mitigates in rats behavioral features that resemble the core and associated symptoms of ASD. Thus, this study suggests that abnormalities in anandamide activity may underlie the deleterious impact of environmental risk factors on ASD-relevant behaviors, and that the endocannabinoid system may be a therapeutic target for the core and associated symptoms displayed by autistic patients.
