# Supplementary material for: Targeting anandamide metabolism rescues core and associated autistic-like symptoms in rats prenatally exposed to valproic acid
Source: Transl Psychiatry. 2016 Sep 27;6(9):e902–. doi: 10.1038/tp.2016.182 (PMC5048215; doi:10.1038/tp.2016.182)
Supplement: Supplementary Figure 1 [file tp2016182x1.ppt]

## Slide 1
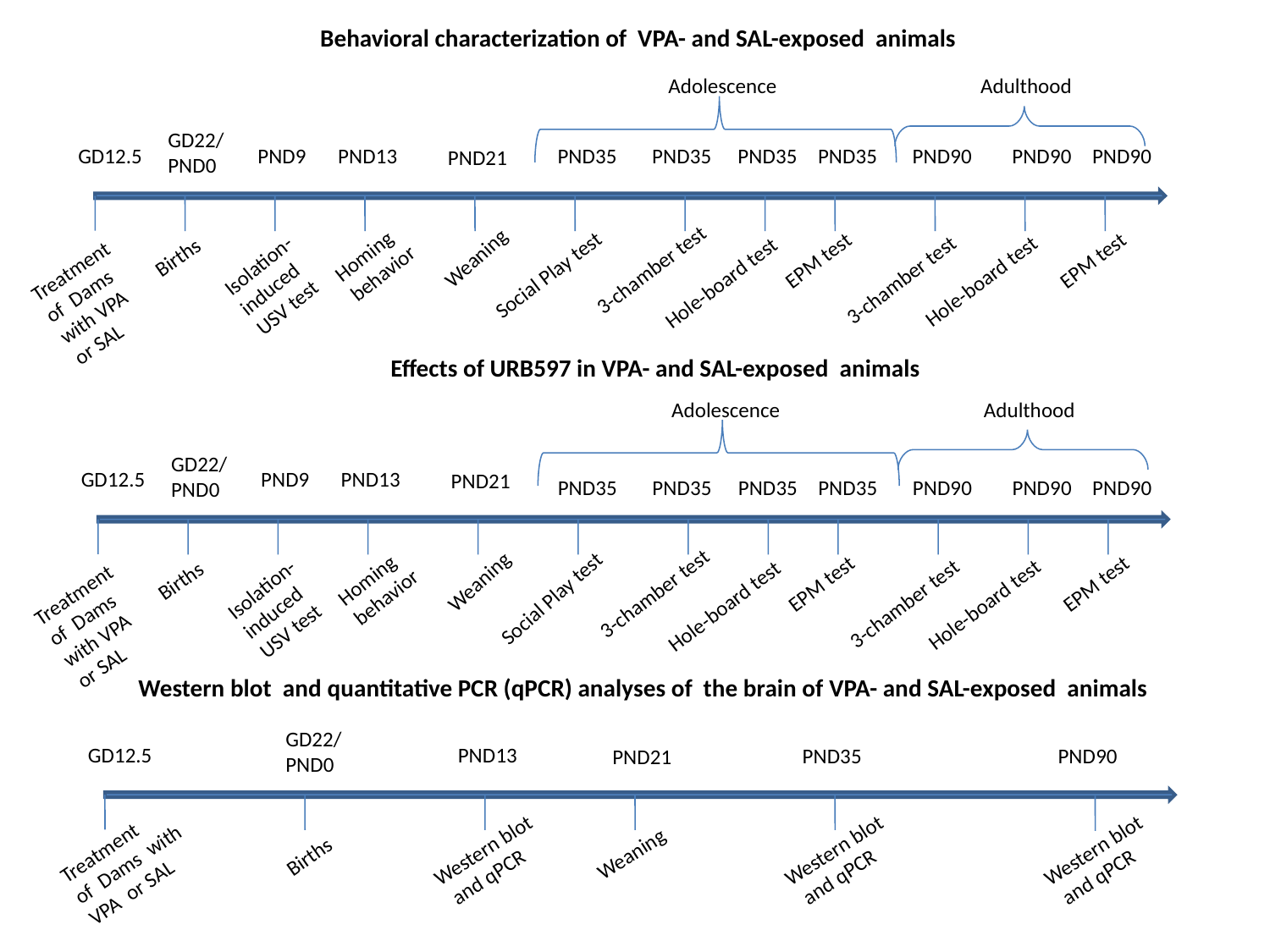

Behavioral characterization of VPA- and SAL-exposed animals
Adolescence
Adulthood
GD22/
PND0
GD12.5
PND9
PND13
PND35
PND35
PND35
PND35
PND90
PND90
PND90
PND21
Isolation-induced
USV test
Weaning
Homing
behavior
Treatment
of Dams
with VPA
or SAL
Births
EPM test
EPM test
3-chamber test
Social Play test
3-chamber test
Hole-board test
Hole-board test
Effects of URB597 in VPA- and SAL-exposed animals
Adolescence
Adulthood
GD22/
PND0
GD12.5
PND9
PND13
PND21
PND35
PND35
PND35
PND35
PND90
PND90
PND90
Isolation-induced
USV test
Weaning
Treatment
of Dams
with VPA
or SAL
Homing
behavior
Births
EPM test
EPM test
3-chamber test
Social Play test
3-chamber test
Hole-board test
Hole-board test
Western blot and quantitative PCR (qPCR) analyses of the brain of VPA- and SAL-exposed animals
GD22/
PND0
GD12.5
PND13
PND35
PND90
PND21
Treatment
of Dams with VPA or SAL
Weaning
Western blot
 and qPCR
Western blot
 and qPCR
Western blot
 and qPCR
Births
